# 2-Bromo-4-chloro-6-[(*E*)-*p*-tolyl­imino­meth­yl]phenol

**DOI:** 10.1107/S1600536809003912

**Published:** 2009-02-11

**Authors:** Xinli Zhang

**Affiliations:** a Department of Chemistry, Baoji University of Arts and Science, Baoji, Shaanxi 721007, People’s Republic of China

## Abstract

The mol­ecule of the title compound, C_14_H_11_BrClNO, displays an *E* configuration with respect to the imine C=N double bond. The two aromatic rings are essentially coplanar, forming a dihedral angle of 7.9 (2)°. An intra­molecular O—H⋯N hydrogen bond stabilizes the crystal structure.

## Related literature

For the role of Schiff base ligands in catalysis and electron transfer in living organisms, see: Ueno *et al.* (2006 ▶).
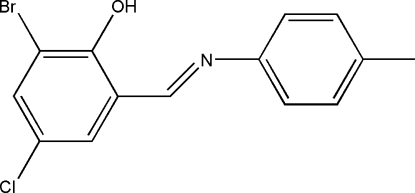


## Experimental

### 

#### Crystal data

C_14_H_11_BrClNO
*M* *_r_* = 324.60
Triclinic, 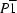

*a* = 8.1354 (14) Å
*b* = 8.6844 (17) Å
*c* = 11.3740 (18) Å
α = 76.040 (2)°
β = 73.652 (12)°
γ = 62.458 (12)°
*V* = 677.9 (2) Å^3^
*Z* = 2
Mo *K*α radiation
μ = 3.22 mm^−1^
*T* = 298 (2) K
0.43 × 0.18 × 0.09 mm

#### Data collection

Siemens SMART CCD area-detector diffractometer
Absorption correction: multi-scan (*SADABS*; Siemens, 1996 ▶) *T*_min_ = 0.332, *T*_max_ = 0.745
3500 measured reflections
2351 independent reflections
1412 reflections with *I* > 2σ(*I*)
*R*_int_ = 0.028

#### Refinement

*R*[*F* ^2^ > 2σ(*F* ^2^)] = 0.045
*wR*(*F* ^2^) = 0.129
*S* = 1.00
2351 reflections
164 parameters
H-atom parameters constrained
Δρ_max_ = 0.51 e Å^−3^
Δρ_min_ = −0.43 e Å^−3^

### 

Data collection: *SMART* (Siemens, 1996 ▶); cell refinement: *SAINT* (Siemens, 1996 ▶); data reduction: *SAINT*; program(s) used to solve structure: *SHELXS97* (Sheldrick, 2008 ▶); program(s) used to refine structure: *SHELXL97* (Sheldrick, 2008 ▶); molecular graphics: *SHELXTL* (Sheldrick, 2008 ▶); software used to prepare material for publication: *SHELXTL*.

## Supplementary Material

Crystal structure: contains datablocks I, global. DOI: 10.1107/S1600536809003912/rz2287sup1.cif

Structure factors: contains datablocks I. DOI: 10.1107/S1600536809003912/rz2287Isup2.hkl

Additional supplementary materials:  crystallographic information; 3D view; checkCIF report

## Figures and Tables

**Table 1:** Hydrogen-bond geometry (Å, °)

*D*—H⋯*A*	*D*—H	H⋯*A*	*D*⋯*A*	*D*—H⋯*A*
O1—H1⋯N1	0.82	1.84	2.574 (4)	148

**Fig. 1.**
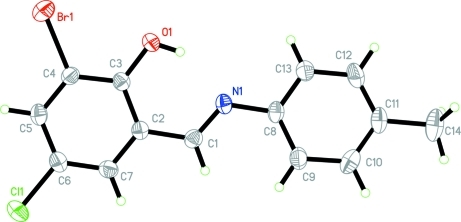
The molecular structure of the title compound with 30% probability ellipsoids. H atoms are shown as spheres of arbitrary radii. The dashed line represents a hydrogen bond.

**Fig. 2.**
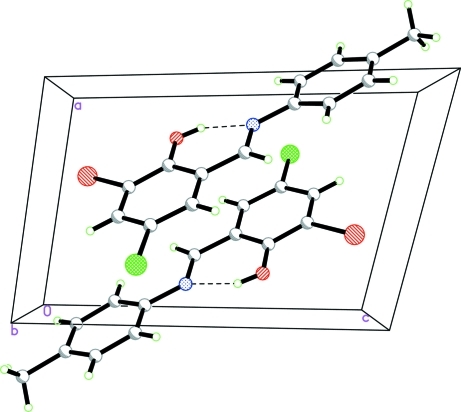
The crystal packing of the title compound viewed along the b axis.

## supplementary crystallographic information

### Comment

Recently, there has been a growing interest in Schiff base ligands because of their applications, such as catalysts and non-linear optical materials. In recent years, they were found to play an important role in the catalysis and electron transfer of the living organisms (Ueno *et al.*, 2006). This stimulated our interest in this field. As an extension of the work on the structural characterization of Schiff base compounds, the crystal structure of the title compound is reported here.

The molecular structure and crystal packing of the title compound are illustrated in Figure 1 and 2, respectively. Bond lengths and angles are not unusual, with the C1═N1 bond distance (1.263 (5) Å) slightly shorter than a normal C═N. The molecule is essentially planar, the maximum deviation from the planarity being 0.167 (6) Å for atom C10. The dihedral angle between the two aromatic rings is 7.9 (2) °. An intramolecular O—H···N hydrogen bond (Table 1) stabilizes the crystal structure.

### Experimental

3-Bromo-5-chlorosalicylaldehyde (0.1 mmol, 23.6 mg) and *p*-toluidine (0.1 mmol, 10.7 mg) were dissolved in methanol (10 ml). The mixture was stirred at room temperature for 10 min and then filtered. After allowing the filtrate to stand in air for 3 d, yellow block-shaped crystals of the title compound suitable for X-ray analysis were formed by slow evaporation of the solvent. The crystals were collected, washed with methanol and dried in a vacuum desiccator using anhydrous CaCl_2_ (yield 54%).

### Refinement

All H atoms were placed in geometrically idealized positions and constrained to ride on their parent atoms, with C—H = 0.93-0.96 Å, O—H = 0.82 Å, and with *U*_iso_(H) = 1.2 *U*_eq_(C) or 1.5 *U*_eq_(C, O) for methyl and hydroxy H atoms.

### Crystal data

**Table:**

C_14_H_11_BrClNO	*Z* = 2
*M**_r_* = 324.60	*F*(000) = 324
Triclinic, *P*1	*D*_x_ = 1.590 Mg m^−^^3^
Hall symbol: -P 1	Mo *K*α radiation, λ = 0.71073 Å
*a* = 8.1354 (14) Å	Cell parameters from 1148 reflections
*b* = 8.6844 (17) Å	θ = 2.7–24.9°
*c* = 11.3740 (18) Å	µ = 3.22 mm^−^^1^
α = 76.040 (2)°	*T* = 298 K
β = 73.652 (12)°	Block-shaped, yellow
γ = 62.458 (12)°	0.43 × 0.18 × 0.09 mm
*V* = 677.9 (2) Å^3^	

### Data collection

**Table:**

Siemens SMART CCD area-detector diffractometer	2351 independent reflections
Radiation source: fine-focus sealed tube	1412 reflections with *I* > 2σ(*I*)
graphite	*R*_int_ = 0.028
φ and ω scans	θ_max_ = 25.0°, θ_min_ = 1.9°
Absorption correction: multi-scan (*SADABS*; Siemens, 1996)	*h* = −9→9
*T*_min_ = 0.332, *T*_max_ = 0.745	*k* = −6→10
3500 measured reflections	*l* = −13→13

### Refinement

**Table:**

Refinement on *F*^2^	Primary atom site location: structure-invariant direct methods
Least-squares matrix: full	Secondary atom site location: difference Fourier map
*R*[*F*^2^ > 2σ(*F*^2^)] = 0.045	Hydrogen site location: inferred from neighbouring sites
*wR*(*F*^2^) = 0.129	H-atom parameters constrained
*S* = 1.00	*w* = 1/[σ^2^(*F*_o_^2^) + (0.065*P*)^2^] where *P* = (*F*_o_^2^ + 2*F*_c_^2^)/3
2351 reflections	(Δ/σ)_max_ < 0.001
164 parameters	Δρ_max_ = 0.51 e Å^−^^3^
0 restraints	Δρ_min_ = −0.42 e Å^−^^3^

### Special details

**Table:**

Geometry. All e.s.d.'s (except the e.s.d. in the dihedral angle between two l.s. planes) are estimated using the full covariance matrix. The cell e.s.d.'s are taken into account individually in the estimation of e.s.d.'s in distances, angles and torsion angles; correlations between e.s.d.'s in cell parameters are only used when they are defined by crystal symmetry. An approximate (isotropic) treatment of cell e.s.d.'s is used for estimating e.s.d.'s involving l.s. planes.
Refinement. Refinement of *F*^2^ against ALL reflections. The weighted *R*-factor *wR* and goodness of fit *S* are based on *F*^2^, conventional *R*-factors *R* are based on *F*, with *F* set to zero for negative *F*^2^. The threshold expression of *F*^2^ > σ(*F*^2^) is used only for calculating *R*-factors(gt) *etc*. and is not relevant to the choice of reflections for refinement. *R*-factors based on *F*^2^ are statistically about twice as large as those based on *F*, and *R*- factors based on ALL data will be even larger.

### Fractional atomic coordinates and isotropic or equivalent isotropic displacement parameters (Å^2^)

**Table:**

	*x*	*y*	*z*	*U*_iso_*/*U*_eq_	
Br1	0.62666 (9)	0.48664 (8)	0.11313 (5)	0.0881 (3)	
Cl1	0.2620 (2)	1.09756 (16)	0.31821 (13)	0.0714 (4)	
O1	0.7924 (4)	0.3591 (4)	0.3427 (3)	0.0554 (9)	
H1	0.8367	0.3272	0.4054	0.083*	
N1	0.8449 (5)	0.3835 (5)	0.5491 (3)	0.0414 (9)	
C1	0.7224 (6)	0.5406 (6)	0.5417 (4)	0.0434 (11)	
H1A	0.6902	0.6050	0.6058	0.052*	
C2	0.6298 (6)	0.6243 (6)	0.4356 (4)	0.0381 (10)	
C3	0.6708 (6)	0.5270 (6)	0.3398 (4)	0.0387 (10)	
C4	0.5770 (6)	0.6130 (6)	0.2422 (4)	0.0439 (11)	
C5	0.4522 (6)	0.7843 (6)	0.2351 (4)	0.0457 (11)	
H5	0.3916	0.8380	0.1683	0.055*	
C6	0.4168 (6)	0.8776 (6)	0.3294 (4)	0.0467 (11)	
C7	0.5034 (6)	0.7974 (6)	0.4289 (4)	0.0476 (11)	
H7	0.4763	0.8608	0.4925	0.057*	
C8	0.9370 (6)	0.3016 (6)	0.6518 (4)	0.0416 (11)	
C9	0.9271 (7)	0.3898 (7)	0.7423 (4)	0.0553 (13)	
H9	0.8552	0.5102	0.7394	0.066*	
C10	1.0267 (7)	0.2956 (8)	0.8377 (4)	0.0619 (14)	
H10	1.0201	0.3549	0.8981	0.074*	
C11	1.1339 (7)	0.1177 (7)	0.8446 (4)	0.0541 (13)	
C12	1.1407 (7)	0.0343 (7)	0.7539 (4)	0.0604 (14)	
H12	1.2122	−0.0862	0.7571	0.072*	
C13	1.0454 (6)	0.1229 (6)	0.6584 (4)	0.0522 (12)	
H13	1.0540	0.0623	0.5981	0.063*	
C14	1.2405 (8)	0.0205 (8)	0.9481 (4)	0.0813 (18)	
H14A	1.2169	0.1011	1.0021	0.122*	
H14B	1.3735	−0.0336	0.9144	0.122*	
H14C	1.1990	−0.0679	0.9938	0.122*	

### Atomic displacement parameters (Å^2^)

**Table:**

	*U*^11^	*U*^22^	*U*^33^	*U*^12^	*U*^13^	*U*^23^
Br1	0.1111 (6)	0.0784 (5)	0.0686 (4)	−0.0123 (4)	−0.0436 (4)	−0.0269 (3)
Cl1	0.0773 (9)	0.0392 (7)	0.0848 (9)	−0.0057 (7)	−0.0321 (8)	−0.0064 (6)
O1	0.058 (2)	0.0406 (19)	0.0586 (19)	−0.0026 (17)	−0.0275 (16)	−0.0109 (15)
N1	0.037 (2)	0.042 (2)	0.046 (2)	−0.0165 (19)	−0.0143 (17)	0.0014 (17)
C1	0.047 (3)	0.046 (3)	0.042 (2)	−0.023 (3)	−0.010 (2)	−0.005 (2)
C2	0.036 (2)	0.041 (3)	0.043 (2)	−0.021 (2)	−0.010 (2)	−0.002 (2)
C3	0.033 (2)	0.039 (3)	0.046 (2)	−0.015 (2)	−0.011 (2)	−0.004 (2)
C4	0.043 (3)	0.047 (3)	0.042 (2)	−0.015 (2)	−0.011 (2)	−0.011 (2)
C5	0.042 (3)	0.048 (3)	0.048 (3)	−0.020 (2)	−0.017 (2)	0.004 (2)
C6	0.045 (3)	0.042 (3)	0.053 (3)	−0.020 (2)	−0.015 (2)	0.002 (2)
C7	0.051 (3)	0.040 (3)	0.055 (3)	−0.018 (2)	−0.015 (2)	−0.009 (2)
C8	0.034 (2)	0.052 (3)	0.041 (2)	−0.023 (2)	−0.012 (2)	0.004 (2)
C9	0.056 (3)	0.053 (3)	0.055 (3)	−0.019 (3)	−0.024 (2)	0.002 (2)
C10	0.062 (3)	0.087 (4)	0.052 (3)	−0.040 (3)	−0.019 (3)	−0.008 (3)
C11	0.049 (3)	0.068 (4)	0.047 (3)	−0.031 (3)	−0.020 (2)	0.016 (3)
C12	0.061 (3)	0.047 (3)	0.069 (3)	−0.021 (3)	−0.029 (3)	0.016 (3)
C13	0.053 (3)	0.048 (3)	0.055 (3)	−0.018 (3)	−0.019 (2)	−0.003 (2)
C14	0.080 (4)	0.110 (5)	0.061 (3)	−0.052 (4)	−0.037 (3)	0.028 (3)

### Geometric parameters (Å, °)

**Table:**

Br1—C4	1.885 (4)	C7—H7	0.9300
Cl1—C6	1.732 (5)	C8—C13	1.381 (6)
O1—C3	1.329 (5)	C8—C9	1.389 (6)
O1—H1	0.8200	C9—C10	1.399 (6)
N1—C1	1.263 (5)	C9—H9	0.9300
N1—C8	1.425 (5)	C10—C11	1.374 (7)
C1—C2	1.462 (5)	C10—H10	0.9300
C1—H1A	0.9300	C11—C12	1.372 (7)
C2—C7	1.372 (6)	C11—C14	1.506 (6)
C2—C3	1.411 (5)	C12—C13	1.376 (6)
C3—C4	1.387 (5)	C12—H12	0.9300
C4—C5	1.357 (6)	C13—H13	0.9300
C5—C6	1.386 (6)	C14—H14A	0.9600
C5—H5	0.9300	C14—H14B	0.9600
C6—C7	1.370 (6)	C14—H14C	0.9600
			
C3—O1—H1	109.5	C13—C8—N1	116.6 (4)
C1—N1—C8	122.3 (4)	C9—C8—N1	124.4 (4)
N1—C1—C2	121.9 (4)	C8—C9—C10	119.3 (5)
N1—C1—H1A	119.0	C8—C9—H9	120.4
C2—C1—H1A	119.0	C10—C9—H9	120.4
C7—C2—C3	120.0 (4)	C11—C10—C9	121.8 (5)
C7—C2—C1	120.0 (4)	C11—C10—H10	119.1
C3—C2—C1	119.9 (4)	C9—C10—H10	119.1
O1—C3—C4	120.8 (4)	C12—C11—C10	117.6 (4)
O1—C3—C2	121.9 (4)	C12—C11—C14	122.0 (5)
C4—C3—C2	117.3 (4)	C10—C11—C14	120.4 (5)
C5—C4—C3	122.8 (4)	C11—C12—C13	122.2 (5)
C5—C4—Br1	118.7 (3)	C11—C12—H12	118.9
C3—C4—Br1	118.5 (3)	C13—C12—H12	118.9
C4—C5—C6	118.7 (4)	C12—C13—C8	120.2 (5)
C4—C5—H5	120.7	C12—C13—H13	119.9
C6—C5—H5	120.7	C8—C13—H13	119.9
C7—C6—C5	120.5 (4)	C11—C14—H14A	109.5
C7—C6—Cl1	120.9 (4)	C11—C14—H14B	109.5
C5—C6—Cl1	118.5 (3)	H14A—C14—H14B	109.5
C6—C7—C2	120.6 (4)	C11—C14—H14C	109.5
C6—C7—H7	119.7	H14A—C14—H14C	109.5
C2—C7—H7	119.7	H14B—C14—H14C	109.5
C13—C8—C9	119.0 (4)		
			
C8—N1—C1—C2	179.4 (4)	Cl1—C6—C7—C2	−178.2 (3)
N1—C1—C2—C7	−177.3 (4)	C3—C2—C7—C6	−0.4 (6)
N1—C1—C2—C3	2.6 (6)	C1—C2—C7—C6	179.5 (4)
C7—C2—C3—O1	179.9 (4)	C1—N1—C8—C13	170.2 (4)
C1—C2—C3—O1	0.1 (6)	C1—N1—C8—C9	−11.1 (6)
C7—C2—C3—C4	−0.8 (6)	C13—C8—C9—C10	−0.2 (7)
C1—C2—C3—C4	179.3 (4)	N1—C8—C9—C10	−178.8 (4)
O1—C3—C4—C5	−179.8 (4)	C8—C9—C10—C11	−0.1 (7)
C2—C3—C4—C5	1.0 (6)	C9—C10—C11—C12	0.1 (7)
O1—C3—C4—Br1	−0.2 (6)	C9—C10—C11—C14	179.5 (4)
C2—C3—C4—Br1	−179.5 (3)	C10—C11—C12—C13	0.2 (7)
C3—C4—C5—C6	0.1 (7)	C14—C11—C12—C13	−179.2 (4)
Br1—C4—C5—C6	−179.5 (3)	C11—C12—C13—C8	−0.5 (7)
C4—C5—C6—C7	−1.3 (6)	C9—C8—C13—C12	0.5 (7)
C4—C5—C6—Cl1	178.4 (3)	N1—C8—C13—C12	179.2 (4)
C5—C6—C7—C2	1.4 (7)		

### Hydrogen-bond geometry (Å, °)

**Table:**

*D*—H···*A*	*D*—H	H···*A*	*D*···*A*	*D*—H···*A*
O1—H1···N1	0.82	1.84	2.574 (4)	148
